# Combined anterior cruciate ligament reconstruction and lateral meniscal root repair yields low failure rates, but inferior subjective outcomes compared to isolated anterior cruciate ligament reconstruction: A comparative study of independent tunnel and anterior cruciate ligament bone tunnel techniques

**DOI:** 10.1002/jeo2.70405

**Published:** 2025-09-04

**Authors:** Christoffer von Essen, Riccardo Cristiani, Björn Barenius, Anders Stålman

**Affiliations:** ^1^ Department of Molecular Medicine and Surgery, Section of Sports Medicine Karolinska Institutet Stockholm Sweden; ^2^ Stockholm Sports Trauma Research Center (SSTRC) FIFA Medical Centre of Excellence Stockholm Sweden

**Keywords:** ACL reconstruction, functional outcome, lateral meniscus root tear, meniscal repair, meniscus failure

## Abstract

**Purpose:**

To assess the functional and subjective outcomes of combined anterior cruciate ligament reconstruction (ACLR) and lateral meniscal root tear (LMRT) repair. Additionally, to compare the use of an independent tunnel for LMRT repair with the ACL bone tunnel technique and to assess the failure rates of LMRT repair to isolated ACLR.

**Methods:**

Patients who underwent primary ACLR and concomitant LMRT repair from May 2017 to May 2022 at Capio Artro Clinic, Stockholm, Sweden, were retrospectively identified and matched 1:3 (age, sex and graft type) with patients who underwent isolated ACLR during the same period. Functional outcomes, including range of motion (ROM), anterior knee laxity and isokinetic strength, were assessed preoperatively and at 6 months, and the knee injury and osteoarthritis outcome score (KOOS) at 2 years. Failure of LMRT repair was defined as the need for reoperation with meniscal resection during the follow‐up period.

**Results:**

A total of 84 patients were included (mean age 31.1 ± 11.1 years; 61.9% male). LMRT repair failure occurred in 7.1% of cases. No significant differences were observed between tunnel techniques in ROM, laxity, or strength. At 2 years, KOOS scores were significantly lower in the ACLR + LMRT group compared to the isolated ACLR group in pain (86.1 ± 15.2 vs. 91.3 ± 13.3, *p* = 0.039), symptoms (79.3 ± 19.1 vs. 86.3 ± 17.3, *p* = 0.017), sport (69.2 ± 26.4 vs. 82.1 ± 25.3, *p* = 0.017) and quality of life (61.2 ± 25.3 vs. 75.3 ± 26.2, *p* = 0.03). Only 29.2% of patients in the LMRT group achieved a patient acceptable symptom state (PASS), compared to 65.2% in the isolated ACLR group (*p* < 0.01).

**Conclusion:**

Combined ACLR and LMRT repair resulted in a 7.1% failure rate. However, the addition of LMRT repair results in lower subjective outcomes compared to isolated ACLR. The use of an independent tibial tunnel for LMRT repair does not confer additional clinical benefit over the shared ACL tunnel technique.

**Level of Evidence:**

Level III.

**Abbreviations**
ACL: anterior cruciate ligament
ACLR: anterior cruciate ligament reconstruction
ADL: activities of daily living
BTB: bone‐patellar tendon‐bone
IKDC: International Knee Documentation Committee
KOOS: knee injury and osteoarthritis outcome score
KOOS4: mean score of the koos pain, symptoms, sports/recreation and quality of life subscales
LMRT: lateral meniscal root tear
LSI: limb symmetry index
MMRT: medial posterior meniscus root tear
MRT: meniscus root tear
PASS: patient acceptable symptom state
ROM: range of motion
SD: standard deviation
SKLR: Swedish Knee Ligament Register
TF: treatment failure

## INTRODUCTION

Meniscus root tears (MRTs) are defined as radial tears located within one centimetre of the root attachment or as a complete avulsion of the meniscus from its tibial attachment [4]. While medial posterior meniscus root tears (MMRTs) are most commonly described as degenerative injuries, frequently observed in older or obese patients with minimal or no associated trauma [14], lateral meniscal root tears (LMRTs) are more commonly traumatic and frequently occur in conjunction with anterior cruciate ligament (ACL) injuries. The reported incidence of LMRTs in ACL‐injured knees is up to 18% [16, 17].

Biomechanical studies indicate that the consequences of an untreated LMRT are similar to those of a total meniscectomy. The consequences include a loss of meniscal load‐sharing capacity, increased contact pressures on the lateral compartment, and a heightened risk of cartilage degeneration. Although the lateral meniscus plays a smaller role in load transmission than the medial meniscus, the long‐term consequences of untreated LMRTs remain a concern, as the progression to osteoarthritis may be somewhat slower [3, 7, 8]. Given these findings, meniscal repair should be strongly considered whenever feasible to restore joint mechanics and minimise long‐term degenerative changes [34].

The transtibial pullout repair technique is the most commonly used method for LMRT repair [3, 6]. This can be performed using either a separate tibial tunnel or the same tunnel used for ACL reconstruction (shared tunnel technique). While both techniques aim to restore the anatomical footprint of the meniscal root, the clinical relevance of tunnel configuration remains unclear. Independent tunnel drilling may offer more precise anatomical placement but carries risks such as tunnel convergence and increased surgical complexity [10, 23].

Despite the biomechanical rationale for LMRT repair, clinical evidence remains limited [5, 30]. Most studies are small, lack control groups, or do not compare surgical techniques. Furthermore, few have evaluated whether LMRT repair affects outcomes when performed alongside ACL reconstruction, or how these outcomes compare to isolated ACLR.

The purpose of the present study was to assess the functional and subjective outcomes of combined anterior cruciate ligament reconstruction (ACLR) and LMRT repair. Additionally, to compare the use of an independent tunnel for LMRT repair with the ACL bone tunnel technique and to assess the failure rates of LMRT repair to isolated ACLR. It was hypothesised that (1) LMRT repair performed in conjunction with ACLR results in inferior subjective outcomes compared to isolated ACLR, and (2) the use of an independent tibial tunnel for LMRT repair does not confer superior functional or subjective outcomes compared to the shared ACL tunnel technique.

## METHODS

Patients who underwent primary ACLR and concomitant LMRT repair from May 2017 to May 2022 at Capio Artro Clinic, Stockholm, Sweden, were identified. All the patients included had a healthy contralateral knee. Patients with associated ligament injuries were excluded. Surgical records and patient characteristics, including age, sex, activity at injury and any reoperation were reviewed. Failure of LMRT repair was defined as the need for reoperation with meniscal resection during the follow‐up period due to persistent clinical symptoms and confirmed intraoperatively. Asymptomatic failures were not assessed, as no routine imaging or second‐look arthroscopy was performed in asymptomatic patients.

For the comparative analysis, each patient treated with ACLR and concomitant LMRT repair was matched with three patients from The Swedish Knee Ligament Register (SKLR) who had undergone ACLR without any concomitant injury. The matching terms were: age, sex and graft type, with matching patients selected with surgery during the inclusion period. Follow‐up data were not part of the matching procedure.

This study was conducted and reported in accordance with the STROBE (Strengthening the Reporting of Observational Studies in Epidemiology) guidelines for observational cohort studies.

### Surgical technique and rehabilitation

Surgical procedures were performed by 12 fellowship‐trained surgeons. Standard arthroscopic portals were utilised. ACLR was performed using a single‐bundle technique. Hamstring, bone‐patellar tendon‐bone, or quadriceps tendon autografts were used. LMRT repair was performed using the transtibial pull‐out technique. Either a separate transtibial tunnel was drilled using a meniscal root guide, exiting at the anatomical root insertion site, or the drilled ACL tibial tunnel was utilised according to the surgeon's preference. Looped sutures (Fiberlink, Arthrex) were passed through the meniscal root under arthroscopic visualisation using a suture‐passing device (Knee Scorpion, Arthrex) creating cinch stitches. The sutures were retrieved through the tunnel and secured over either an AO bicortical screw with a washer or a SwiveLock (Arthrex) anchor for cortical fixation.

All patients adhered to a standardised rehabilitation protocol, using a hinged brace for a total of 6 weeks, with an increase of 30° of flexion every 2 weeks (0–30° first and second weeks, 0–60° third and fourth and 0–90° fifth and sixth weeks) and no during this period.

### Patient evaluation

Range of motion (ROM) and anterior knee laxity were measured preoperatively and at 6 months after the ACLR. A goniometer was used for measuring ROM, and loss of ROM was defined as >5° loss of extension and flexion compared to the contralateral healthy knee. The KT‐1000 arthrometer (MEDmetric) with a standard anterior tibial load of 134N at 20° of knee flexion was used to measure anterior tibial translation. Laxity was reported stratified according to the IKDC form.

At 6 months, isokinetic knee extension and flexion strength was measured bilaterally at 90°/s using the Biodex System 3 (Biodex Medical Systems) and reported as limb symmetry index (LSI). The LSI is the percentage difference in strength between the injured and uninjured limb, using the uninjured limb as a reference.

The knee injury and osteoarthritis outcome score (KOOS) [29] was collected preoperatively and at a 2‐year follow‐up. The KOOS evaluates patients' perception of knee function on five subscales (pain, symptoms, activities of daily living, sports and recreation and quality of life), with scores ranging from 0 (worst) to 100 (best). The KOOS4 is a mean score of four subscales, in which the ADL subscale is excluded to avoid a ceiling effect, as relatively young and active patients seldom have difficulties with ADL [12]. To interpret the outcome of KOOS4, the patient acceptable symptom state (PASS) and treatment failure were applied [28]. The PASS for KOOS4 is a validated threshold indicating whether patients consider their knee function satisfactory, while treatment failure marks patients who feel that the treatment has failed. In this study, PASS was applied using the thresholds by Roos et al. to determine the proportion of patients who achieved a satisfactory outcome [28].

Comparisons were also made for KOOS domain and KOOS4 between the patients undergoing ACLR with or without LMRT at 2 years two treatment with greedy matching (ACLR LMRT: ACLR) for age, sex and graft using a nearest neighbour approach [2].

Each LMRT‐treated patient was matched against three patients who had undergone ACLR during the same time period.

### Statistical analysis

Statistical analysis was performed using the SPSS software package (version 25.0, IBM Corp.). Demographic characteristics were reported using descriptive statistics. Normal distribution of the data was assessed using the Shapiro–Wilk test. Group comparisons were made using the chi‐square test for categorical variables, Student's *t*‐test for continuous variables, and Fisher's exact test for counts expected to be smaller than five. To make a comparison for the KOOS subscales, TF, and PASS between ACL‐injured patients without any meniscal damages, a greedy matching (ACLR: ACLR with LMRT) for age, gender and graft was made, using a nearest neighbour approach. The minimum level of significance was set to *p* = 0.05.

## RESULTS

**Figure 1:**
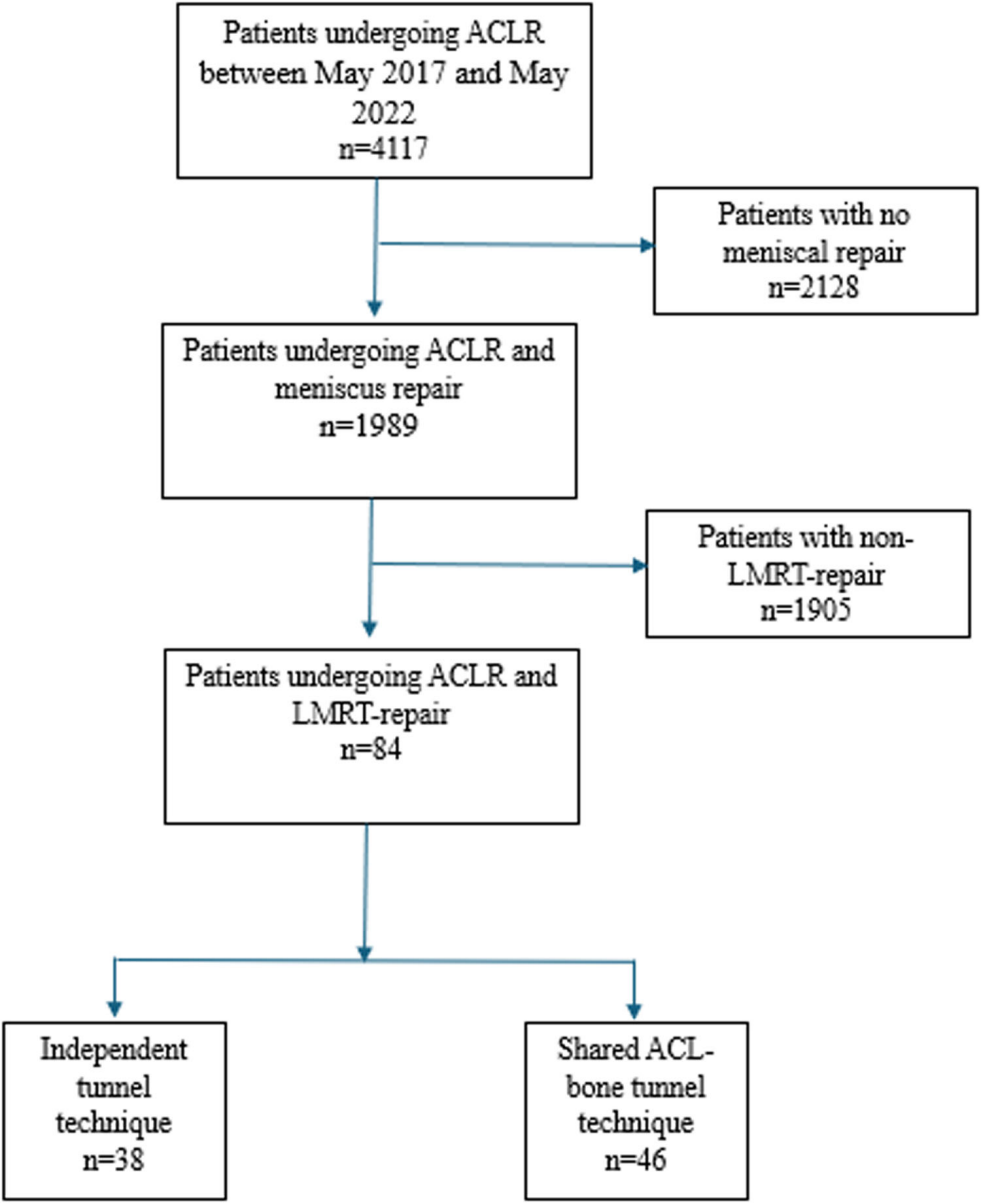
Patients' flowchart. ACL, anterior cruciate ligament; ACLR, anterior cruciate ligament reconstruction; LMRT, lateral meniscus root tear.

In total, 84 patients were eligible for inclusion. In 38 patients (45.2%), a separate tunnel was created and in 46 patients (54.8%) the repair was done through a shared ACL bone tunnel (Figure 1). The mean age at surgery across the cohort was 31.1 ± 11.1 years and 52 patients (61.9%) were male. The minimum follow‐up time was 2 years, and the mean follow‐up time was 45 ± 12 months. Patient demographics are presented in Table 1.

**Table 1:** Patient demographics, intraoperative characteristics and LMRT‐failures.

	Independent tibial tunnel technique *n* = 38	Shared ACL bone tunnel technique *n* = 46	*p* value
Age at surgery (mean ± SD)	31.1 ± 11.1	31.0 ± 11.1	n.s.
Gender			
Male	26 (68.4)	26 (56.5)	n.s.
Graft type			
Hamstring tendon	22 (57.9)	26 (56.5)	n.s.
Quadriceps tendon	16 (42.1)	17 (36.9)	
BTB	0	3 (6.5)	
Failure of meniscus	2 (5.3)	4 (8.7)	n.s.

*Note*: Data are reported as *n* (%) unless otherwise indicated.

Abbreviations: ACL, anterior cruciate ligament; BTB, bone‐patellar tendon‐bone; LMRT, lateral meniscal toot tear; SD, standard deviation.

During the follow‐up time, six patients (7.1%) suffered failure of the LMRT repair. Six patients (7.1%) underwent revision ACLR, seven patients (8.3%) underwent subsequent medial meniscal procedure and eight patients (9.5%) underwent surgery for a cyclops lesion. No surgical‐technique‐related complication with the LMRT repair was reported.

### ROM and laxity

Preoperative and 6 months postoperative ROM and anterior knee laxity, as well as 6 months of isokinetic knee extension and flexion strength measurements, were not significantly different between the study groups (Table 2).

**Table 2:** ROM, laxity preoperative and at 6 months, flexion and extension LSI at 6 months.

	Total	Independent tibial tunnel technique	Shared ACL bone tunnel technique	*p* value
ROM				
Pre‐operative	*n* = 49	*n* = 21	*n* = 28	
Flexion deficit >5°	1 (2.0)	0	1 (3.6)	n.s.
Extension deficit >5°	7 (14.3)	1 (4.8)	6 (21.4)	n.s.
Missing	35 (41.7)	17 (44.7)	18 (39.1)	
6 months	*n* = 53	*n* = 24	*n* = 29	
Flexion deficit >5°	6 (11.5)	4 (17.4)	2 (6.9)	n.s.
Extension deficit >5°	5 (9.4)	4 (16.7)	1 (3.4)	n.s.
Missing	31 (36.9)	14 (36.8)	17 (36.9)	
Laxity				
Pre‐operative	*n* = 56	*n* = 26	*n* = 30	
≤2 mm	32 (57.1)	15 (57.7)	17 (56.7)	n.s.
3–5 mm	15 (26.8)	6 (23.1)	9 (30.0)	n.s.
>5 mm	9 (16.1)	5 (19.2)	4 (13.3)	n.s.
Missing	28 (33.3)	12 (31.6)	16 (34.8)	
6 months	*n* = 27	*n* = 11	*n* = 16	
≤2 mm	22 (81.4)	9 (81.8)	13 (81.3)	n.s.
3–5 mm	5 (18.5)	2 (18.2)	3 (18.8)	n.s.
>5 mm		‐	‐	
Missing	57 (67.9)	27 (71.1)	30 (65.2)	
Strength LSI >90%				
6 months	*n* = 32	*n* = 14	*n* = 18	
Extension	6 (18.8)	4 (28.6)	2 (11.1)	n.s.
Flexion	20 (62.5)	12 (85.7)	8 (44.4)	0.03
Missing	52 (61.9)	24 (63.2)	28 (60.9)	

*Note*: Data are reported as *n* (%) unless otherwise indicated.

Abbreviations: ACL, anterior cruciate ligament; LSI, limb symmetry index; ROM, range of motion.

### Subjective knee function

At the 2‐year follow‐up, 12 patients (29.2%) achieved a PASS, considering their outcome as good, while five patients (12.2%) were TF (Table 3). There was a significant improvement in KOOS4 at 2 years.

**Table 3:** Mean KOOS values and proportion of patients achieving PASS and TF at the 2‐year follow‐up.

	Independent tibial tunnel technique *n* = 38	Shared ACL bone tunnel technique *n* = 46	*p* value
KOOS, mean ± SD			
Pain	85.1 ± 16.2	87.1 ± 15.2	n.s.
Symptoms	80.3 ± 20.2	78.3 ± 19.1	n.s.
ADL	94.4 ± 13.3	94.4 ± 11.3	n.s.
Sport	67.3 ± 25.3	75.4 ± 28.2	n.s.
QoL	57.1 ± 27.4	63.2 ± 25.4	n.s.
Treatment failure	3 (21.4)	2 (7.4)	n.s.
PASS KOOS4	3 (21.4)	9 (33.3)	n.s.
Missing	24 (63.2)	19 (41.3)	

Abbreviations: ACLR, anterior cruciate ligament reconstruction; ADL, activities of daily living; KOOS, knee injury and osteoarthritis outcome score; KOOS4, mean score of the knee injury and osteoarthritis outcome score pain, symptoms, sports/recreation and quality of life subscales; PASS, patient‐acceptable symptom state; QoL, quality of life; TF, treatment failure.

Demographic characteristics of patients who underwent ACLR and LMRT and isolated ACLR are reported in Table 4.

**Table 4:** Demographic characteristics of patients in the isolated ACLR and ACLR and LMRT repair groups.

	ACLR and LMRT‐repair *n* = 84	Isolated ACLR *n* = 252
Age at surgery, mean ± SD	31.1 ± 11.1	31.1 ± 11.1
Gender		
Male	52 (61.9)	156 (61.9)
Graft type		
Hamstring tendons	48 (57.1)	144 (57.1)
Quadriceps tendon	33 (39.2)	99 (39.2)
BTB	3 (3.6)	9 (3.6)

*Note*: Data are reported as *n* (%) unless otherwise indicated.

Abbreviations: ACLR, anterior cruciate ligament reconstruction; BTB, bone‐patellar tendon‐bone; LMRT, lateral meniscal root tear.

**Figure 2:**
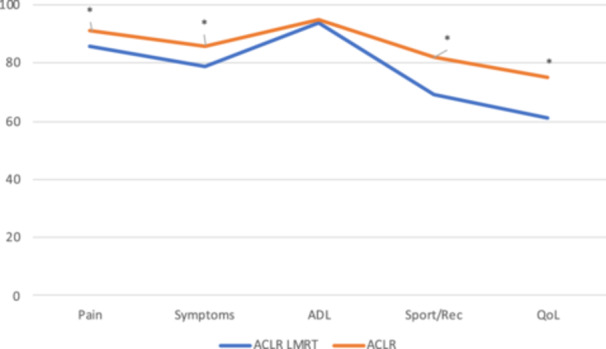
Mean knee injury and osteoarthritis outcome score score for both groups. ACLR, anterior cruciate ligament reconstruction; ADL, activities of daily living; LMRT, lateral meniscal root tear; QoL, quality of life.

At the 2‐year follow‐up, the isolated ACLR group reported significantly higher KOOS scores in all subscales except for ADL subscale in comparison with the ACLR plus LMRT repair group. In addition, the isolated ACLR group had a higher proportion of patients achieving a PASS in comparison with the ACLR plus LMRT repair group (Table 5, Figure 2).

**Table 5:** Mean KOOS scores and proportion of patients achieving PASS and TF at the 2‐year follow‐up.

	ACLR and LMRT repair *n* = 84	Isolated ACLR *n* = 252	*p* value
KOOS, mean ± SD			
Pain	86.1 ± 15.2	91.3 ± 13.3	0.039
Symptoms	79.3 ± 19.1	86.3 ± 17.3	0.017
ADL	94.1 ± 11.4	95.2 ± 12.1	n.s.
Sport	69.2 ± 26.4	82.1 ± 25.3	0.017
QoL	61.2 ± 25.3	75.3 ± 26.2	0.03
Treatment failure	5 (12.2)	11 (6.2)	n.s.
PASS KOOS4	12 (29.2)	116 (65.2)	<0.01
Missing	43 (51.1)	74 (29.4)	

*Note*: Data are reported as *n* (%) unless otherwise indicated.

Abbreviations: ACLR, anterior cruciate ligament reconstruction; ADL, activities of daily living; KOOS, knee injury and osteoarthritis outcome score; KOOS4, mean score of the knee injury and osteoarthritis outcome score pain, symptoms, sports/recreation and quality of life subscales; PASS, patient‐acceptable symptom state; QoL, quality of life; TF, treatment failure.

## DISCUSSION

The most important finding of the present study was that combined ACLR and LMRT repair resulted in a low failure rate. Additionally, LMRT repair with independent tunnel drilling did not result in superior objective or subjective outcome compared with shared ACLR tibial tunnel repair. However, combined ACLR and LMRT repair resulted in a significantly lower subjective outcome compared with isolated ACLR. These findings support the hypotheses stated in the introduction: (1) that LMRT repair in conjunction with ACLR would result in inferior subjective outcomes compared to isolated ACLR and (2) that the use of an independent tibial tunnel would not provide superior outcomes compared to the shared ACL tunnel technique.

Among the 84 patients included in the study, six (7.1%) had LMRT repair failure. This is similar to a recent systematic review including 215 patients reporting a healing rate above 90% after LMRT repair in combination with ACLR [35]. Ahn et al. [1] performed 25 side‐to‐side LMRT repair with concomitant ACLR and found complete healing in eight out of nine patients at second‐look arthroscopy. Similarly, LaPrade et al. [23] reported no surgical failures at 2 years in 14 patients who underwent LMRT repair. In a recent study by De Leissègues et al. including 99 patients, in which the used both transtibial pull‐out sutures and side‐to‐side LMRT repair, only one patient suffered failure of the repair [5].

Transtibial pullout‐repair is a commonly used technique for LMRT repair which restores the biomechanical properties of the meniscus [21]. The LMRT attachment centre is located 6–11 mm outside the tibial attachment of the posterolateral ACL bundle [15]. Forkel and Petersen introduced the shared ACL bone tunnel LMRT repair technique, allowing the sutures from the LMRT repair to pass through the same tunnel as the ACL graft [10]. As the anatomical attachment of the LMR is close to the ACL tibial attachment, the establishment of an additional independent tunnel can be difficult, prolong surgical time and there is a risk of tunnel collision [13]. A cadaveric experiment has suggested that an independent tunnel might not be necessary [9]. However, Laprade et al. [20] have demonstrated that nonanatomical LMRT repairs do not restore the contact area and increase contact pressures. The present study could not find any significant differences in subjective or objective outcomes between the two techniques.

The incidences of LMRT in association with ACL tears has been reported to be between 7% and 14% in imaging‐based studies [8, 18]. These studies did not differentiate between LMRT that needed repair and stable lesions that could be treated nonoperatively. The sensitivity of MRI is limited [22] and in a study by Krych et al., the authors reported that 67% of LMRTs were missed [19]. The arthroscopic incidence of LMRTs has been reported around 6.7% by Ahn et al. [1] and around 6.6% by Praz et al. [26]. In the present study, the incidence of LMRT‐repairs was a 2.0%. The discrepancy might lead to the conclusion that the LMRTs often are considered stable or can be missed. While the effect of a medial meniscal root tear is meniscal extrusion and osteoarthritis, the consequences of an untreated LMRT is still unclear. The meniscofemoral ligaments and popliteus tendon can both protect the LMRT from extrusion [25]. Shelbourne et al. reported only mild joint space narrowing after 10 years when LMRTs are left in situ [31]. However, in a more recent study by Tsujii et al. [34], the authors reported almost no radiologic changes after repairing the LMRT at a mean 3.4 years.

LaPrade et al. described in a biomechanical study that a LMRT increases anterior and rotatory instability and may increase loads on the ACL graft [11], but the clinical importance of this remains unclear. In another cadaveric study [32], LMRT‐repair reduced anterior knee laxity. This was also seen in the results of this study, where knee laxity was restored in a vast majority of the patients with no significant difference between the two techniques.

A recent systematic review reported improvements in Lysholm and IKDC scores for patients who underwent LMRT repair in combination with ACLR [24]. Similarly, in a case series of 26 patients following LMRT repair in combination with ACLR by Zhou et al. [36] both IKDC and Lysholm scores improved significantly at the final follow‐up. De Leissègues et al. [5] followed up 99 patients with ACLR and LMRT‐repair, and 91 (91.9%) reported their surgical outcome to be ‘good’ or ‘very good’, with a significant improvement. These findings are in line with the results of this study, where a significant improvement in knee function was demonstrated.

In a recent study by Therrien et al. [33], 50 patients with a LMRT‐repair were matched 1:1 with 50 patients who underwent isolated ACLR. The two groups reported comparable, high IKDC scores (92.5 ± 6.8 vs. 91.9 ± 8.2, respectively) and no failures of the LMRT‐repairs. This is not consistent with the findings of our study, where at the final follow‐up significantly lower KOOS scores were reported for the LMRT‐repair group across all subscales except the ADL subscale in comparison with the isolated ACLR group. In addition, patients who underwent ACLR + LMRT repair achieved a PASS to a lower degree compared with patients who underwent isolated ACLR. In the ACLR + LMRT repair group, only 29.2% achieved the threshold level for the PASS, which is significantly lower than the average ACLR reported in the Swedish Knee Ligament Registry [27]. Even if the LMRT‐repair group improved with surgery, a great proportion did not reach an acceptable knee function regarding KOOS. The inferior outcomes observed in the ACLR + LMRT repair group may be attributed to several factors. These include the greater initial injury severity, more conservative rehabilitation protocols, and potential residual biomechanical deficits. Additionally, psychological factors related to undergoing a more complex procedure may influence patient‐reported outcomes.

This study has several limitations. First, it is a retrospective cohort study, which inherently carries a risk of selection bias. The decision to perform LMRT repair and the choice of surgical technique were not randomised and may have been influenced by surgeon preference or intraoperative findings. Furthermore, the control group was drawn from a national registry, which may differ in unmeasured ways from the LMRT cohort. These differences could contribute to the observed disparities in patient‐reported outcomes and should be interpreted with caution. Second, no magnetic resonance imaging or second‐look arthroscopy was performed to evaluate healing. Third, the relatively low follow‐up rates for clinical and functional outcomes may introduce selection bias and limit the generalisability of our findings. Patients with better compliance or outcomes may be overrepresented, potentially skewing the results. Finally, the lack of a control group with untreated LMRTs limits the ability to directly compare outcomes between patients who underwent LMRT repair and those who did not.

## CONCLUSION

Combined ACLR and LMRT repair resulted in a 7.1% failure rate. However, the addition of LMRT repair results in lower subjective outcomes compared to isolated ACLR. The use of an independent tibial tunnel for LMRT repair does not confer additional clinical benefit over the shared ACL tunnel technique.

## AUTHOR CONTRIBUTIONS

**Christoffer von Essen:** Conceptualisation; data processing; statistical analysis; methodology; manuscript writing. **Anders Stålman, Riccardo Cristiani** and **Björn Barenius**: Methodology; critical review of the manuscript. All authors commented on previous versions of the manuscript. All authors read and approved the final manuscript.

## CONFLICT OF INTEREST STATEMENT

The authors declare no conflicts of interest.

## ETHICS STATEMENT

Ethical permission for this study was obtained from the Regional Ethics Committee of Karolinska Institute (Diarie number: 2016/1613‐31/2).

## Data Availability

The data that support the findings of this study are available on request from the corresponding author. The data are not publicly available due to privacy or ethical restrictions.
